# Fear of COVID-19: the mediation role between the COVID-19 diagnosis and KAP in Spanish university students

**DOI:** 10.1186/s12889-023-16777-3

**Published:** 2023-10-03

**Authors:** Ana Cancela, Mar González-Noriega, Ana Visiers

**Affiliations:** 1 Departamento de Psicología, Universidad Villanueva, c/ Costa Brava, 6, Madrid, 28034 Spain; 2 Universidad Europea del Atlántico, Santander, Spain

**Keywords:** Knowledge, Attitudes and Practices, Fear of COVID-19, Diagnosis of COVID-19, Spain, University students

## Abstract

**Background:**

Although health public services recommend prevention strategies for COVID-19 some of these recommendations have not been taken seriously by young people. Understanding why some people comply with these recommendations and others do not seem to be crucial in helping public health services to predict behavior and compliance with rules, especially for young people. Previous studies suggest that knowledge, attitudes, and practices (KAP) are useful to assess compliance with the preventive measures and public health policies. Being afraid has also been found to correlate with more engagement with preventive measures. This study aims to assess the KAP and fear of COVID-19 of Spanish university students and to understand the relation between diagnosis, KAP and the level of fear.

**Method:**

Participants of this cross-sectional study were 598 college students (69.4% women) from different Spanish Universities. Data were collected for a month using an online questionnaire through Sphinx iQ2.

**Results:**

Levels of KAP among Spanish students were satisfactory and results suggest the presence of fear among them. More importantly, fear of COVID-19 mediated the impact of the diagnosis on the KAP.

**Conclusions:**

Feeling fear seems to be the mechanism underlying the relationship between diagnosis and KAP. Diagnosis is associated with KAP when the diagnosis it is accompanied by measures of fear. KAP, diagnosis, or perceived fear of COVID must be taken together in consideration for health interventions and public health campaigns design.

## Background

The coronavirus SARS-CoV-2, responsible for the COVID-19 epidemic, was first identified in Wuhan (China) in December 2019 and the World Health Organization (WHO) recognized a global pandemic on 11 March 2020. At of 30 August 2023, the number of COVID-19 confirmed cases reported by WHO has been 770,085,713, including 6,956,173 deaths. Europe has seen 275,912,918 confirmed cases, of which 13,980,340 are in Spain [1]. In Spain, the first cases were identified on January 31, 2020 [2].

The Spanish Government implemented on March 14, 2020, a general lockdown period with a stay-at-home requirement. That state of alarm was maintained with a second state which ended on 9 May 2021 [3]. In this case the free movement of citizens was limited between hours, territories and number of people who can meet.

More than one million university students were confined when the state of alarm started in Spain. Students began online education, distance learning and digital instruction at the university from March 2020, until the end of the academic year in July 2020. Between September 2020 to July 2021, a hybrid and flexible model (face-to-face instruction and distance learning) started in all Spanish Universities [4].

Although The Centers for Disease Control and Prevention (CDC) and governments of different countries recommend prevention strategies for COVID-19, such as social distancing, there is evidence that some of these recommendations have not been taken seriously by adolescent and young adult (AYA) populations [5–11]. In Spain, adherence to the preventive strategies has been found to be associated to age. People under 45 years old were less likely to follow the acceptance and adherence to the main preventive measures, such as wearing a mask, washing their hands, and keeping distance [12]. These results have also been found in other countries [13–16].

Previous studies suggest that knowledge, attitudes, and practices (KAP) are useful to assess compliance with preventive measures [16–18]. Indeed, health interventions and public health policies have been designed based on KAP surveys outcomes [19]. Although there are studies that have showed consistence between students KAP [6, 20–25] some others have not found this association between knowledge and attitudes or practices [19, 26, 27].

Knowledge seems to help to identify why some people comply with the measures and others do not. In addition, knowledge provides information on which people interventions should be aimed at [16]. A greater adherence to preventive strategies is associated with a higher risk perception [18, 27–30]. However, individuals that are aware to some extent of the risk of COVID-19 typically underestimate their personal risk relative to that of others [18]. In this sense, young people reported lower high-risk perception than adults [11].

Being afraid has been found to be associated with risk perception [29] and to correlate with more engagement with preventive measures [27, 28, 30]. Although this last relationship between fear and preventive measures has not always been found [10]. Fear can be defined as an emotional response to a real or imagined danger causing physiological changes in the body to rise an escape or defensive behavior [29, 31].

Fear of COVID-19 has been found among the population during the pandemic [32–36]. Specifically in Spain, university students do not report particularly high scores of fears, although data suggest the presence of fear [37]. Older people, those with a lower level of education and those belonging to the most socioeconomically vulnerable group showed higher scores in fear [29]. Moreover, one’s own infection or having a person in immediate surroundings infected by COVID-19 (i.e., family member, friend, coworker) increases the probability of fear [29, 38].

Understanding why some people comply with preventive measures and others do not appears to be crucial in helping public health services establishing meaningful predictors of population behavior and compliance, especially for young people. This study aims to assess the knowledge, attitude, behavior (KAP) and fear of COVID-19 of Spanish university students to the COVID‑19 outbreak. In addition, an attempt was made to understand the relation between the diagnosis and the knowledge, attitudes, and practices. Fear of COVID-19 can be a potential mechanism by which diagnosis can produce changes on knowledge, attitudes, and practices.

## Method

### Participants and design

Participants of this cross-sectional study were 598 (69.4% women, *M*_*age*_ = 21.9, *SD* = 4.7) college students from different Spanish Universities. The proportion of responses was 0.03% of the total population of Spanish college students (1,679,518 students on that academic year) [39].

### Procedure

The study data were collected for a month using an online questionnaire through *Sphinx* iQ2 and was shared via social media applications (Facebook, LinkedIn, and WhatsApp) to reach the study population. Also, the link was sent to the Students Department of all Spanish universities to be shared with their students by email. Data were collected over 4 weeks from April 3 to May 9 of 2021, one year after the lockdown measures and movement restrictions were implemented.

All information regarding the study, participant’s rights, and researcher’s contact details were provided on the first page of the survey questionnaire. Information about the study objectives and the procedure was given to the participants followed by their consent to participate. Once they consented to participate, they could access the rest of the questionnaire. None of the incomplete data would be used in the analyses.

### Research instruments / measurements

The survey questionnaire, tailored specifically for this study, had 6 sections. Section 1: brief about the background and the need for the survey. Section 2: informed written consent. Section 3: questions about sociodemographic variables (sex and age). Section 4: eight questions regarding exposure to COVID-19 and related vulnerability issues. Section 5: regarding KAP towards COVID-19 (2 items about knowledge (symptoms and spreading), 18 items about attitudes and 8 items about practices). The total score of the participants’ knowledge about COVID-19 symptoms ranged from 0 to 17 (greater score indicates more knowledge) and a cut off level of ≥ 9 was set for more accurate knowledge. Participants also selected all items they considered to be a COVID-19 spread vias. The total score ranged from 0 to 4 (greater score indicates more knowledge) and a cut off level of ≥ 3 was set. The attitude section assessed beliefs and perceptions towards the COVID-19 pandemic, including the risk of transmission and symptom development (4 items), and the perceived effectiveness of risk reduction strategies (14 items). Responses of each item was indicated on a 5-point Likert scale (1 = *completely disagree*, 5 = *completely agree*). Ratings between items referring to the same issue were inter-correlated, so they were averaged to create 2 composite indexes: perceived own risk of transmission and symptom development (3 items; α = 0.71); perceived risk of spreading (1 item); perceived effectiveness of risk reduction strategies (14 items; α = 0.84). The index was scored such that higher numbers indicate more favorable attitudes toward the topics. Practices assessment included 8 items regarding strategies to control COVID-19 outbreak. The strategies were about hygiene-related behaviors and avoidance-related behaviors. Participants were asked how much they used the risk reduction strategies in a 5-point Likert scale (1 = *never*, 2 = *rarely*, 3 = *sometimes*, 4 = *often*, 5 = *every time/ always*). Ratings were inter-correlated (α = 0.79), so they were averaged to create a composite index. The index was scored such that higher numbers indicate more adaptative behavior regarding risk reduction strategies. Section 6: The Fear of COVID-19 Scale (FCV-19 S [32]; validated Spanish university students’ version [37]).

### Statistical analysis

Frequencies and percentages for categorical variables, measures of central tendency and dispersion were calculated. Student’s t and ANOVA tests were used to determine the relation between means of knowledge, attitudes, and practices scores and socio-demographic variables. Mediation analyses were used to evaluate how being diagnosed with COVID 19 can change knowledge, attitudes, or practices scores. All data analyses were performed using Statistical Package for the Social Sciences (SPSS) software, version 22 and the PROCESS add-on for SPSS [40]. A value of *p* < .05 was considered statistically significant.

## Results

### Exposure to COVID-19 and related vulnerability issues

Table 1 shows the responses to the exposure to COVID-19 and related vulnerability issues.

**Table 1:** Frequencies and percentages of exposure to COVID-19 and related vulnerability issues (N = 598)

Exposure and related vulnerability				*N*		%	
Vulnerability							
	Own´s vulnerability						
		No		539		90.1	
		Yes		59		9.9	
	Relative´s vulnerability						
		No		99		16.6	
		Yes		499		83.4	
Exposure to COVID-19							
	Own´s exposure/ diagnosis						
		No		501		83.8	
		Yes		97		16.2	
			Asymptomatic		20		20.6
			Mild symptoms		72		74.2
			Severe symptoms		5		5.2
	Relative´s exposure/ diagnosis						
		No		287		48.0	
		Yes		311		52.0	
			Asymptomatic		32		10.3
			Mild symptoms		167		53.7
			Severe symptoms		71		22.8
			Death		41		13.2
	Close friend´s exposure/ diagnosis						
		No		106		17.7	
		Yes		492		82.3	
			Asymptomatic		93		18.9
			Mild symptoms		372		75.6
			Severe symptoms		26		5.3
			Death		1		0.2

### Knowledge, attitudes, and practices

#### Knowledge about the COVID-19 pandemic

Out of the 598 students, 532 (88.9%) students answered ≥ 9/17 questions correctly about COVID-19 symptoms and 476 (79.6%) students answered ≥ 3/4questions correctly COVID-19 spreading. Knowledge assessment of the participants regarding common symptoms and ways of spreading are shown in Table 2. The total knowledge (symptoms) score ranged from 1 to 17, with a mean of 10.95 ± 3.29. The total knowledge (spread) score ranged from 1 to 4, with a mean of 3.27 ± 0.91.

**Table 2:** Frequencies and percentages of knowledge about COVID-19 among the participants (N = 598)

Knowledge items		Yes		No	
		*N*	%	*N*	%
Common symptoms include					
	Dry cough	517	86.5	81	13.5
	Fever	575	96.2	23	3.8
	Fatigue	532	89.0	66	11.0
	Pneumonia	407	68.1	191	31.9
	Mucus	164	27.4	434	72.6
	Loss of smell	569	95.2	29	4.8
	Loss of taste	570	95.3	28	4.7
	Nasal congestion	196	32.8	402	67.2
	Headache	498	83.3	100	16.7
	Throat pain	331	55.4	267	44.6
	Muscle or joint pain	436	72.9	162	27.1
	Nausea or vomiting	224	37.5	374	62.5
	Diarrhea	346	57.9	252	42.1
	Chills or vertigo	173	28.9	425	71.1
	Shortness of breath	528	88.3	70	11.7
	Loss of appetite	194	32.4	404	67.6
	Chest pressure	284	47.5	314	52.5
COVID-19 spreads by					
	Droplets transmitted in sneezes and coughs (less than 1 m)	284	47.5	314	52.5
	Droplets transmitted by contact with the eyes, nose, mouth	575	96.2	23	3.8
	Droplets deposited on objects	513	85.8	85	14.2
	Aerosols suspended in the air	411	68.7	187	31.3

#### Attitudes

For each question focused on attitude, the statistics of responses from participants are presented in Table 3. Participant’s perceptions about their own risk of acquiring COVID-19 and the perceived impact on their health mean was 2.72 ± 0.80, with a range of 1 to 5, and a symmetrical and mesokurtic distribution. It is important to notice that they believed to be at risk of getting COVID-19 to a greater extent (3.56 ± 0.99) than of developing severe symptoms (2.62 ± 1.01; *t*_(597)_ = 20.44, *p* < .001) or even dying (1.98 ± 1.02; *t*_(597)_ = 31.41, *p* < .001). Perceived risk of spreading the disease was higher (4.02 ± 0.99) than the perceived risk of getting COVID-19 (3.56 ± 0.99); *t*_(597)_ = -10.30, *p* < .001.

**Table 3:** Descriptive Statistics for attitudes towards the COVID-19 pandemic (N = 598)

Attitude items		Mean (*SD*)	Range	Skewness	Kurtosis	α
**Perceived own risk of transmission and symptom development**		2.72 (0.80)	1–5	0.27	0.05	0.71
	I believe I am at risk of getting COVID-19	3.56 (0.99)	1–5	− 0.58	− 0.03	
	I believe I can develop severe symptoms if I get Covid-19	2.62 (1.01)	1–5	0.42	− 0.19	
	I believe I could die if I catch COVID-19	1.98 (1.02)	1–5	0.82	− 0.09	
**Perceived risk of spreading**		4.02 (0.99)	1–5	− 0.98	0.57	
**Perceived effectiveness of risk reduction strategies**		3.76 (0.58)	1.29-5	− 0.39	0.56	0.84
	Use of mask	4.59 (0.75)	1–5	-2.40	6.85	
	Hand hygiene/ Washing hands	4.55 (0.69)	1–5	-1.88	4.61	
	Ventilation of spaces	4.60 (0.66)	1–5	-1.84	3.81	
	Disinfection of spaces	4.45 (0.85)	1–5	-1.72	2.81	
	Limited capacity	4.36 (0.88)	1–5	-1.56	2.33	
	Social distance	4.27 (0.91)	1–5	-1.45	2.14	
	Online education	3.12 (1.30)	1–5	− 0.03	-1.11	
	Limitation of hours in the restoration	2.71 (1.23)	1–5	0.30	− 0.96	
	Curfew	2.54 (1.31)	1–5	0.38	-1.07	
	Closure of sports facilities and services (gyms, sports centers …)	2.66 (1.25)	1–5	0.25	− 0.96	
	Closure of cultural facilities and services (libraries, museums, cinemas …)	2.26 (1.16)	1–5	0.73	− 0.27	
	Home isolation	3.36 (1.35)	1–5	− 0.40	-1.10	
	Diagnostic test	4.59 (0.69)	1–5	-1.94	4.46	
	Vaccine	4.59 (0.82)	1–5	-2.25	5.09	

**Fig. 1:**
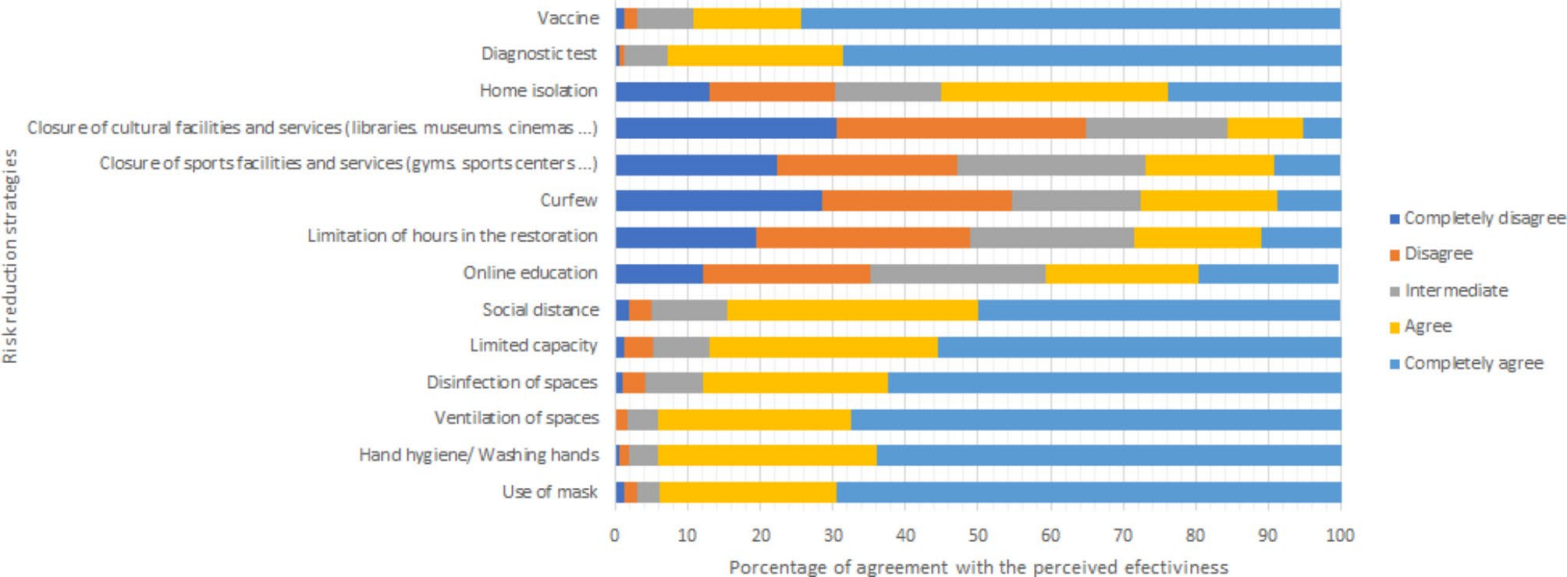
Rating of level of perceived effectiveness of risk reduction strategies

Participants’ attitude towards the degree of effectiveness of measures to reduce personal risk from COVID-19 was satisfactory as the mean attitude score was 3.76 ± 0.58, with a range of 1.29 to 5 (Table 3). Figure 1 shows the perceptions towards the degree of effectiveness of measures to reduce personal risk from COVID-19. Ventilation of spaces and washing hands were considered to have high to very high effectiveness (*N* = 562, 94% and *N* = 562, 94%, respectively), followed by wearing a mask (*N* = 561, 93.8%), taking a diagnostic test (*N* = 554, 92.7%), vaccine (*N* = 533, 89.1%), disinfection of spaces (*N* = 525, 87.8%), limited capacity of places (*N* = 521, 87.1%) and social distance (*N* = 505, 84.4%).

#### Practices

**Fig. 2:**
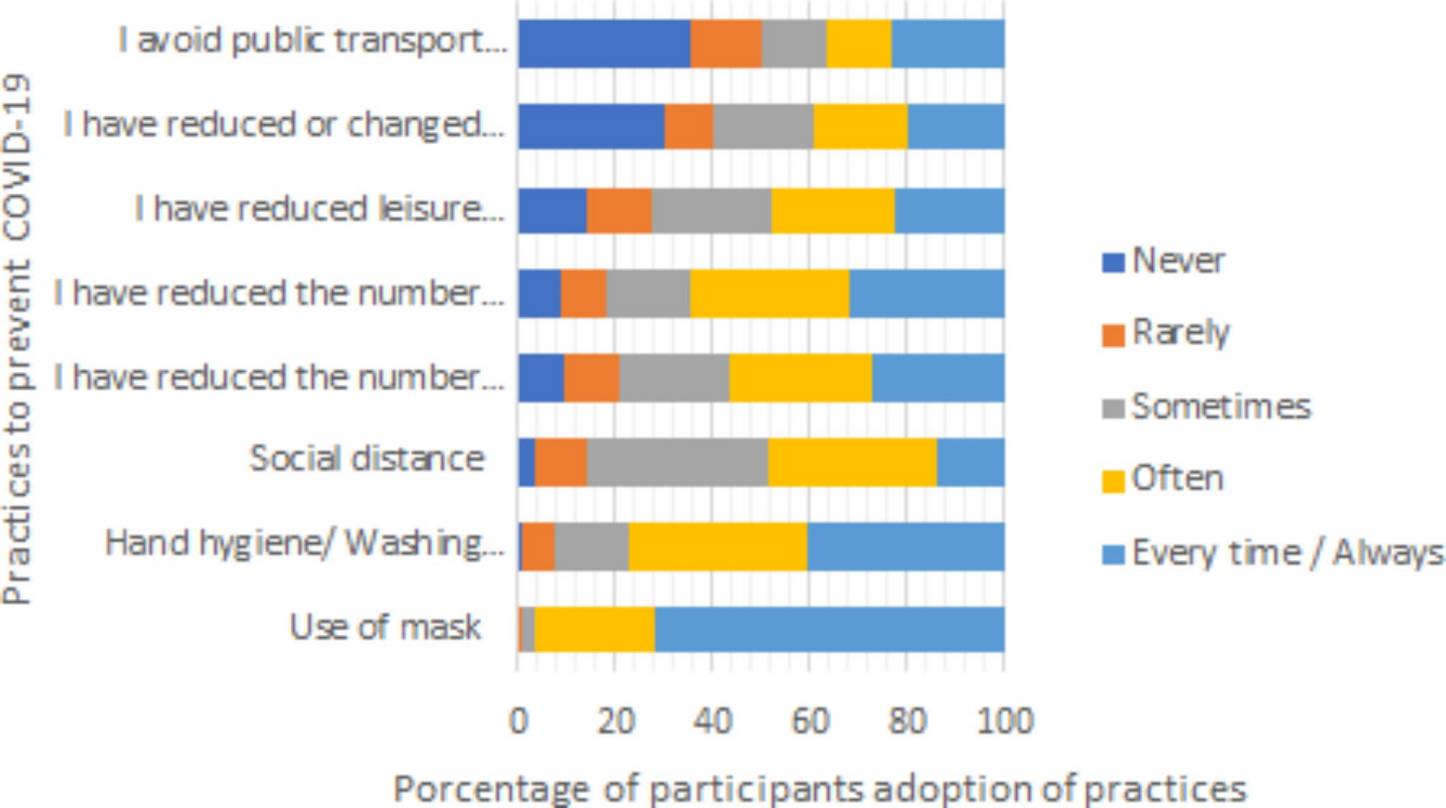
Ratings of participant’s adoption of practices to prevent COVID-19

Participant’s adoption of practices to prevent COVID-19 was satisfactory as the mean practices score was 28.40 ± 6.19, with a range of 11 to 40 (Table 4). Figure 2 shows the degree of adoption of each practice. The most common strategies to control COVID-19 outbreak were the hygiene-related behaviors: 576 (96.3%) students wear often or always a mask and 460 (76.9%) washed their hands often or always. Wearing a mask was mandatory in Spain at the time data were collected. Taking in consideration only the avoidance-related behaviors results showed that the most common strategy was reducing the number of social contacts with family (*N* = 387, 64.7%), followed by reducing the number of social contacts with friends (*N* = 340, 56.9%), keeping social distance (*N* = 293, 48.9%) and avoiding going to bars or public events (*N* = 288, 48.1%).

**Table 4:** Descriptive Statistics for participant’s adoption of practices to prevent COVID-19 (N = 598)

Practices items		Mean (*SD*)	Range	Skewness	Kurtosis	α
**Using risk reduction strategies**		3.55 (0.77)	11–40	− 0.26	− 0.42	0.79
	Use of mask	4.68 (0.57)		-1.92	4.68	
	Hand hygiene/ Washing hands	4.09 (0.94)		− 0.89	0.19	
	Social distance	3.45 (0.98)		− 0.34	− 0.10	
	I have reduced the number of social contacts with friends.	3.54 (1.26)		− 0.56	− 0.68	
	I have reduced the number of social contacts with family.	3.70 (0.25)		− 0.78	− 0.38	
	I have reduced leisure activities: I avoid going to bars or public events.	3.29 (1.34)		− 0.32	-1.02	
	I have reduced or changed the type of sports activities.	2.89 (1.51)		0.02	-1.44	
	I avoid public transport whenever I can.	2.75 (1.61)		0.24	-1.54	

### Fear of COVID-19

Participants’ mean value on the FCV-19 S (α = 0.86) was 15.08 (*SD* = 5.75), median = 14.00, with a range of 7 to 35.

### Relationships between variables

In order to facilitate the analysis, a Knowledge Index was created combining the two items related to knowledge (r =. 27) and an Attitude Index combining the 3 attitudes items related to attitudes (α = 0.51).

There was a positive correlation (*r* = .12) between knowledge and attitudes scores (*p* = .003) and knowledge and practices score (*r* = .13, *p* = .001); attitude and practice scores increased as knowledge scores were increasing. There was also a moderate positive correlation (*r* = .33) between attitude and practices score (*p* < .001); practices scores increased as attitude scores were increasing. This correlation was stronger analyzing only the attitudes toward effectiveness of risk reduction strategies and practices scores (*r* = .49, *p* < .001). As the perceived effectiveness of risk reduction strategies scores increased the adoption of strategies to control COVID-19 scores also increased.

Women, compared to men, reported having more knowledge (*M* = 7.40, *SD* = 1.68), more positive attitudes (*M* = 3.55, *SD* = 0.56) and adopted more risk reduction strategies (*M* = 3.63, *SD* = 0.74) than men (*M* = 6.45, *SD* = 1.96, *t*_(304)_ = -5.69, *p* < .001; *M* = 3.39, *SD* = 0.60, *t*_(596)_ = -3.03, *p* < .001; *M* = 3.36, *SD* = 0.81, *t*_(596)_ = -4.11, *p* < .001; respectively). There was a positive correlation between age and attitudes (*r* = .12, p = .003); as well as a correlation between age and practices scores (*r* = .21, *p* < .001).

Those participants who reported being vulnerable to Covid-19 due to a medical condition reported more positive attitudes (*M* = 3.75, *SD* = 0.59) and adopted more risk reduction strategies (*M* = 3.86, *SD* = 0.78) than those who are less vulnerable (*M* = 3.47, *SD* = 0.57, *t*_(596)_ = -3.56, *p* < .001; *M* = 3.51, *SD* = 0.77, *t*_(596)_ = -3.3, *p* = .001; respectively). Participants having a relative vulnerable to COVID-19 also reported more positive attitudes (*M* = 3.53, *SD* = 0.56) and adopted more risk reduction strategies (*M* = 3.58, *SD* = 0.69) than those who did not (*M* = 3.31, *SD* = 0.63, *t*_(596)_ = -3.52, *p* < .001; *M* = 3.38, *SD* = 0.69, *t*_(153.3)_ = -2.69, *p* = .008; respectively). Those participants who had been diagnosed with COVID-19 reported more knowledge (*M* = 7.63, *SD* = 1.91) and lower positive attitudes (*M* = 3.39, *SD* = 0.56) than those who had not (*M* = 7.00, *SD* = 1.79, *t*_(596)_ = -3.14, *p* = .002; *M* = 3.52, *SD* = 0.58, *t*_(596)_ = 2.05, *p* = .04; respectively). Also, participants who had a relative that had been diagnosed with COVID-19 reported more knowledge (*M* = 7.32, *SD* = 1.75) than those who did not (*M* = 6.88, *SD* = 1.87, *t*_(596)_ = -2.95, *p* = .003).

There was a moderate positive correlation (*r* = .39) between fear of COVID-19 and attitude scores (*p* < .001); attitude scores increased as fear of COVID-19 scores were increasing. There was a moderate positive correlation (*r* = .35) between fear of COVID-19 and practices scores (*p* < .001); practices scores increased as fear of COVID-19 scores were increasing. A small positive correlation (*r* = .22) was observed between fear scores and knowledge scores (*p* < .001).

Women reported having more fear of COVID-19 (*M* = 16.07, *SD* = 5.76) compared to men (*M* = 12.83, *SD* = 5.05, *t*_(394)_ = -6.93, *p* < .001).

There was a positive correlation between age and fear of COVID-19 (*r* = .005, *p* = .90). Level of fear increased as the age increased.

Those participants suffering from any disease that makes them vulnerable to COVID-19 reported having more fear of COVID-19 (*M* = 17.98, *SD* = 1.68) than those who did not (*M* = 14.76, *SD* = 5.51, *t*_(66)_ = -3.44, *p* = .001).

Participants who had been diagnosed with COVID-19 reported less fear of COVID-19 (*M* = 13.96, *SD* = 5.27) than those who had not (*M* = 15.29, *SD* = 5.81, *t*_(596)_ = 2.10, *p* = .04).

There were no differences in the fear of COVID-19 scale between participants who had a relative that had been diagnosed with COVID-19 (*M* = 16.00, *SD* = 5.55) and those who did not (*M* = 15.05, *SD* = 5.95, *t*_(596)_ = − 0.10, *p* = .92). It is important to note that results of the ANOVA of the severity of the relative’s symptoms on fear of COVID-19 were significant, *F*(3,310) = 4.21, *p* = .004. Participants who had suffered the death of a relative from COVID-19 reported significantly more fear (*M* = 17.56, *SD* = 6.69) compared with those whose relatives had passed the illness with mild symptoms (*M* = 14.59, *SD* = 5.02; *p* = .01) or asymptomatic (*M* = 13.41, *SD* = 5.22; *p* = .008).

### Mediation

**Fig. 3:**
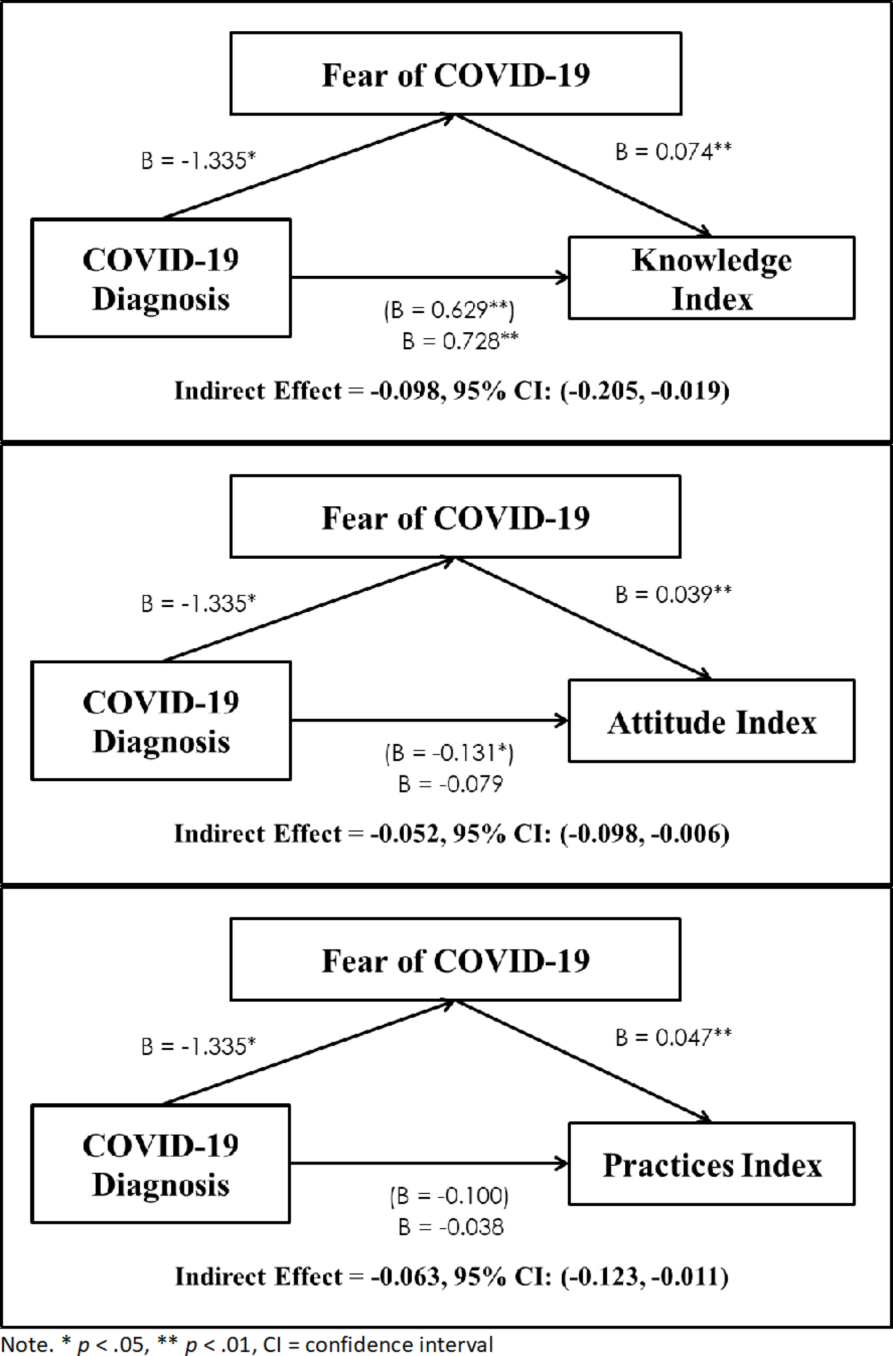
Mediated model predicting KAP as a function of Diagnosis wit Fear of COVID-19 as the meadiating variable

Mediation analysis was conducted to determine whether fear of COVID-19 mediated the relationships between having been diagnosed with COVID-19 and KAP. The unstandardized coefficients for the indirect effects were as follows: β = − 0.09, 95% *CI* [-0.021, − 0.02] for the Knowledge Index; β = − 0.05, 95% *CI* [–0.09, –0.006] for the Attitude Index; β = − 0.06, 95% *CI* [-0.12, − 0.01] for the Practices Index. These results showed that fear of COVID-19 mediated the impact of the diagnosis on the KAP variables (see Fig. 3).

## Discussion

Most of the participants of this study were knowledgeable about COVID-19 like other similar university students’ samples [19, 21, 23, 25, 41]. The participants’ attitude towards the degree of effectiveness of measures to reduce personal risk from COVID-19 was satisfactory. However, participants’ perceptions about their own risk acquiring COVID-19 were lower than the perceived risk of spreading the disease. Moreover, they believe to be at risk of getting COVID-19 to a greater extent than of developing severe symptoms or even dying. These results are coherent with those claiming that, although, individuals can be aware to some extent of the risk of the COVID-19, they typically underestimate their personal risk [18] or were more worried for their family and friends not to get infected than themselves [42]. Participants’ adoption of practices to prevent COVID-19 were also satisfactory as in other university students’ samples [21, 25, 41].

All measures of KAP showed a relationship between them, as knowledge increased so did attitudes and adherence to preventive strategies as in previous studies [6, 20–25, 43]. Also, as attitudes scores increased (i.e., more risk perception and more perception of the preventive strategies effectiveness) more compliance with the strategies was found. Similarly, other studies have been demonstrated that, although knowledge influences the attitudes, the attitudes are the ones that influences practices [44], particularly toward COVID-19 [45]. It is important to notice that the relationship was stronger between the perceived effectiveness of risk reduction strategies and the adoption of strategies to control COVID-19. Coherently, a previous study showed that involvement in more COVID-19 preventive practices was associated with greater COVID-19 perceived risk, in turn, with more COVID-19 fear [46].

Vulnerability was not related to knowledge. However, participants who were more vulnerable or had a vulnerable close person reported more positive attitudes and adopted more risk reduction strategies than those who are less vulnerable themselves or close family or friends.

Results suggest that being diagnosed or having a relative that has been diagnosed with COVID-19 is related to more knowledge. But it does not seem to be related to having more positive attitudes or more engagement with the preventive strategies. Indeed, this study showed that being diagnosed is related to lower attitudes.

Similar to other study carried out in Spain with university students, participants do not inform of high scores of fears, although they suggest the presence of fear [37]. The probability of feeling fear of COVID-19 was related to KAP, for example not maintaining social relation with non-cohabiting people [47]. As previous research had shown [25, 30, 48] participants reporting more knowledge, more positive attitudes or more compliance with preventive strategies also reported feeling more fear. Moreover, higher risk perceptions increased the probability of feeling fear [29]. Being vulnerable or having close vulnerable friends or family members increased the probability of feeling fear as living with a chronically ill family member is related to high negative emotional responses in young people [47].

Although evidence has shown that the probability of fear of infection increases if the person has been infected by COVID-19 or if a close person has been infected [29, 38] results did not show this relationship. Indeed, participants who had been diagnosed with COVID-19, reported less fear of COVID-19. However, when analyzing the data differentiating between the several consequences of the diagnosis, severity of the symptoms of the relative was found to be related to more fear. As in previous studies [38, 49], participants who have suffered the death of a relative from COVID-19 reported significantly more fear, higher anxiety symptom and stress. In this sense, this lack of fear can be explained because most of the sample who had been diagnosed reported being asymptomatic or with mild symptoms. Being asymptomatic or with mild symptoms could also be the explanation of the lower positive attitudes of the students diagnosed with COVID-19 compared who those who had not, despite having a higher knowledge.

The mediation analysis can explain the differences between the results found in this study and previous research. Fear of COVID-19 mediated the impact of the diagnosis on the KAP variables. Feeling fear seems to be the mechanism underlying the relationship between diagnosis and KAP. Diagnosis is associated with KAP when the diagnosis it is accompanied by measures of fear. It is important to take in consideration that high intolerance of uncertainty is one factor that explains COVID-19 fear in the Spanish population [50] and the survey of the present study was conducted in a very uncertain context: media reports were constant, there was a great lack of knowledge about the disease and its long-term consequences; and population vaccination was still limited. In Spain, the vaccination campaign started at the end of December 2020, in old people’s homes and health-care staff. In February the vaccination campaign was offer to other groups of populating organized by age and starting with people over-80s. At the time of the survey only the vulnerable students could have access to the vaccine.

These findings could help public health services in developing effective health promotion campaigns. It is important to improve people attitudes producing a consequent healthy behavior [51]. Also, public health campaigns and doctors when giving the diagnosis could target fear as a useful tool in some situations. Fear produces behavioral change when people feel a sense of efficacy [52]. Increasing KAP could also increase the sense of efficacy. Attitudes regarding the expectations of effectiveness of the measure against COVID-19 and the very use of prevention practices against COVID-19 is in themselves the exit strategy to the threat.

This study has some limitations. First, the questionnaire used has not been validated, which could lead to information bias. Secondly, although we tried to reach all Spanish universities, we failed to do it and thus, this may not be a good representative of all Spanish university students. Because it was an online questionnaire, students without internet connections could not provide their opinions.

## Conclusions

Taking together the results of this study and previous research it seems that health interventions and public health policies cannot only be designed based on KAP surveys outcomes, diagnosis, or perceived fear by themselves. All variables must be taken together in consideration. These findings suggest a need for continuous health education and health promotion to improve adherence to prevention measures for COVID-19 or other pandemics. Future research must focus on the use of fear tactics as a useful tool in a public health campaign to improve the compliance with preventive behaviors in new epidemic contexts.

## Data Availability

The data that support the findings of this study are available from Universidad Villanueva but restrictions apply to the availability of these data, which were used under license for the current study, and so are not publicly available. Data are however available from the authors upon reasonable request to the corresponding author (acancela@villanueva.edu) and with permission of Universidad Villanueva.
